# Antibiotic use for respiratory syncytial virus in the Middle East: A surveillance study in hospitalized Jordanian children

**DOI:** 10.1371/journal.pone.0260473

**Published:** 2021-11-29

**Authors:** Danielle A. Rankin, Nikhil K. Khankari, Zaid Haddadin, Olla Hamdan, Ahmad Yanis, Samir Faouri, Asem Shehabi, John V. Williams, Najwa Khuri-Bulos, Natasha B. Halasa

**Affiliations:** 1 Department of Pediatrics, Vanderbilt University Medical Center, Nashville, Tennessee, United States of America; 2 Vanderbilt Epidemiology PhD Program, Vanderbilt University School of Medicine, Nashville, Tennessee, United States of America; 3 Division of Genetic Medicine, Vanderbilt University Medical Center, Nashville, Tennessee, United States of America; 4 Department of Pediatrics, Al Bashir Hospital, Amman, Jordan; 5 Department of Pediatrics, Jordan University, Amman, Jordan; 6 Department of Pediatrics, University of Pittsburgh School of Medicine, Children’s Hospital of Pittsburgh of University of Pittsburgh Medical Center, Pittsburgh, Pennsylvania, United States of America; Federal University of Sergipe, BRAZIL

## Abstract

**Introduction:**

In developing countries where point-of-care testing is limited, providers rely on clinical judgement to discriminate between viral and bacterial respiratory infections. We performed a cross-sectional cohort study of hospitalized Jordanian children to evaluate antibiotic use for respiratory syncytial virus (RSV) infections.

**Materials and methods:**

Admitting diagnoses from a prior viral surveillance cohort of hospitalized Jordanian children were dichotomized into suspected viral-like, non-pulmonary bacterial-like, and pulmonary bacterial-like infection. Stratifying by sex, we performed a polytomous logistic regression adjusting for age, underlying medical condition, maternal education, and region of residence to estimate prevalence odds ratios (PORs) for antibiotic use during hospitalization. Sensitivity and specificity of admission diagnoses and research laboratory results were compared.

**Results:**

Children with a suspected viral-like admission diagnosis, compared to those with suspected non-pulmonary bacterial-like, were 88% and 86% less likely to be administered an empiric/first-line antibiotic (male, aPOR: 0.12; female, aPOR: 0.14; p-value = <0.001). There were slight differences by sex with males having a lower prevalence than females in being administered an expanded coverage antibiotic; but they had a higher prevalence of macrolide administration than males with non-pulmonary bacterial-like infection. Overall, children with RSV had a 34% probability (sensitivity) of being assigned to a suspected viral-like diagnosis; whereas RSV-negative children had a 76% probability (specificity) of being assigned to a suspected pulmonary bacterial-like diagnosis.

**Conclusions:**

Hospitalized children with a suspected viral-like admission diagnosis were less likely to receive an empiric/first-line and expanded coverage antibiotic compared to suspected non-pulmonary and pulmonary infections; however, when evaluating the accuracy of admission diagnosis to RSV-laboratory results there were considerable misclassifications. These results highlight the need for developing antibiotic interventions for Jordan and the rest of the Middle East.

## Introduction

Respiratory viruses are the leading cause of lower respiratory tract infections in young children worldwide, with respiratory syncytial virus (RSV) as the most common [1]. Symptom-based diagnoses of viral infections, including RSV, are difficult given a similar presentation to pulmonary bacterial infections (i.e., pneumonia) [2]. Common symptoms of both viral and bacterial pulmonary infections include fever, cough, wheezing, and shortness of breath. Excluding influenza, recommended treatment for respiratory viral infections are supportive therapy; however, these respiratory viral infections are commonly misidentified as bacterial pneumonia, which is typically treated empirically with antibiotics [3]. In countries where diagnostic testing is not accessible, clinicians rely on their clinical judgement to discriminate between viral and bacterial infections for determining whether antibiotics are warranted. Exposure to antibiotics for an infection of viral origin poses risks to patients for future adverse drug reactions and acquisition of multidrug resistance [3], which is a global public health threat [4–6] attributable to approximately 35,000 deaths in the United States each year [7].

Patterns of antimicrobial prescriptions are well characterized in the United States; however, few studies have been conducted to assess prescribing practices in the Middle East. In Jordan, only two studies have evaluated antibiotic prescribing among children with respiratory infections. One evaluated the proportions of antibiotics prescriptions to children seen at an emergency department [8]; and the second assessed the frequency and predictors of prescribing practices at several ambulatory care settings [9]. While both studies have provided important information regarding the prescribing patterns for viral diagnoses, neither study confirmed the diagnoses using laboratory testing.

To establish antibiotic stewardship strategies and guidelines for hospitalized pediatric patients in Jordan, additional research on the antibiotic use in diagnostic resource-limited settings are needed. Our primary goal of this study was to evaluate the association of clinical admission diagnoses and antibiotic administration practices in children less than two years who presented with fever and/or respiratory symptoms and were hospitalized at a government-run hospital in Amman, Jordan, where point-of-care viral testing is lacking. Based on our previous research, 64% of children hospitalized in Jordan with a virus had RSV detected in their respiratory specimen [10]. Thus as a secondary analysis we aim to evaluate the accuracy of clinical admission diagnoses compared to RSV-research laboratory testing to inform whether point-of-care viral testing would aid as an antibiotic stewardship tool. We hypothesized those with research laboratory-confirmed RSV are more likely to be administered antibiotics during their hospitalization than children with no RSV detection, due to the similarities of symptoms between bacterial and viral-like pulmonary infections and the high prevalence of RSV in Jordan.

## Materials & methods

### Study design and population

We conducted a cross-sectional cohort study from our main three-year prospective respiratory viral surveillance study at Al-Bashir Hospital, in Amman Jordan (population > 2 million), which is one of three government hospitals in Jordan [10,11]. Children under two years who were hospitalized with acute respiratory symptoms and/or history of fever from March 2010 to March 2013 were included in our cohort. Those never discharged from the hospital or were admitted with fever and neutropenia were excluded and not enrolled into our study [11]. Written informed consent was obtained from parents and/or legal guardian preceding enrollment into our study [11]. Our study was approved by the Institutional Review Boards at the University of Jordan, the Jordan Ministry of Health, and Vanderbilt University [11].

### Data collection

After we obtained informed consent from the parents/guardian of an eligible child, we conducted an interview collecting information on demographics, family history, social history (e.g., number of household members, smoke exposure, siblings, etc.), maternal education, delivery method, prenatal care, and region of residence (i.e., proximity to hospital). Trained research personnel administered interviews in Arabic using a standardized questionnaire and recorded responses in English [10,11]. Medical records were abstracted for each subject after discharge, which included obtaining admission diagnoses, antibiotics, antibiotic duration, and provider-ordered bacterial culture results [10]. Demographic, interview, medical record, and laboratory results were maintained in a secure REDCap^TM^ (Research Electronic Data Capture, Vanderbilt University, Nashville, TN, USA) database [11,12].

### Specimen collection and laboratory methods

Trained research personnel collected a nasal and throat swab; and both were combined into transport medium (M4RT^®^, Remel, USA), aliquoted into MagMAX^TM^ Lysis/Binding Solution Concentrate (Life Technologies, USA), snap-frozen, stored at -70°C, and shipped on dry ice to Nashville, TN, USA [10,11]. We conducted testing of original and lysis buffer aliquots through real-time reverse transcriptase polymerase chain reaction (RT-qPCR) for eleven respiratory viruses, including RSV. Research results were not provided to the clinicians in real-time [10,11].

### Exposure

Our exposure is clinical presentation which was created from a child’s primary admission diagnosis given by the admitting physician. The admission diagnoses were categorized into suspected viral-like infection (i.e., RSV, bronchiolitis, acute respiratory infection, upper respiratory infection, influenza, apnea, asthma, croup, and wheezing) pulmonary bacterial-like infection (i.e., pertussis, pneumonia, and bronchopneumonia), and non-pulmonary bacterial-like infection (i.e., sepsis, febrile, febrile seizure, tonsillitis [referent]). Children with a both a symptom diagnosis (e.g., wheezing or apnea) and a condition diagnosis (e.g., bronchopneumonia or sepsis), were categorized by their condition status. Those with conflicting or no admitting diagnoses were excluded from the analysis.

As many admission diagnoses can be of viral and/or bacterial origin, we categorized children with an admission diagnoses that may warrant empiric antibiotic therapy into either the suspected non-pulmonary or pulmonary bacterial-like illness group. Our reasoning for this classification was to emulate the challenges physicians in Jordan face without laboratory diagnostic testing.

### Outcome

The primary outcome of our study is defined as the administration of at least one antibiotic throughout the individual’s hospitalization course. Antibiotics were categorized into four levels: 1) None administered; 2) Empiric/first-line (i.e., ampicillin, gentamicin, cefotaxime, ceftriaxone, cefuroxime); 3) Macrolides (i.e., erythromycin, clarithromycin, and azithromycin); and 4) Expanded coverage (i.e., vancomycin, ceftazidime, and amikacin). The administration of antibiotics were retrospectively collected through medical chart abstractions for all enrolled children.

### Statistical analysis

Differences in demographic and clinical characteristics of children were evaluated using simple linear regression with robust standard errors for continuous variables and Pearson χ^2^ test for categorical variables. Potential confounders were identified using *a priori* knowledge and the 10% change-in-estimate criterion. We evaluated potential effect measure modification by sex. Statistically significant differences (*p-value* <0.20) in each stratum were evaluated on the multiplicative scale with the likelihood ratio test. Polytomous logistic regression adjusting for spline-coded age, underlying medical conditions, maternal education, and region of residence were conducted to estimate the prevalence odds ratios (aPORs) and 95% confidence intervals (CIs) for the administration of empiric/first-line, macrolides, or expanded coverage antibiotics during hospitalization (compared to no antibiotic administration). Sensitivity and specificity were calculated to evaluate the accuracy of admission diagnosis in classifying the causative pathogen (i.e., RSV-positive versus RSV-negative) and further applied to virus-positive vs. virus-negative. Statistical significance was based on two-tailed tests with α = 5%. All analyses were conducted using statistical software StataIC 16.0 (StatCorp LLC, College Station, TX).

## Results

**Fig 1:**
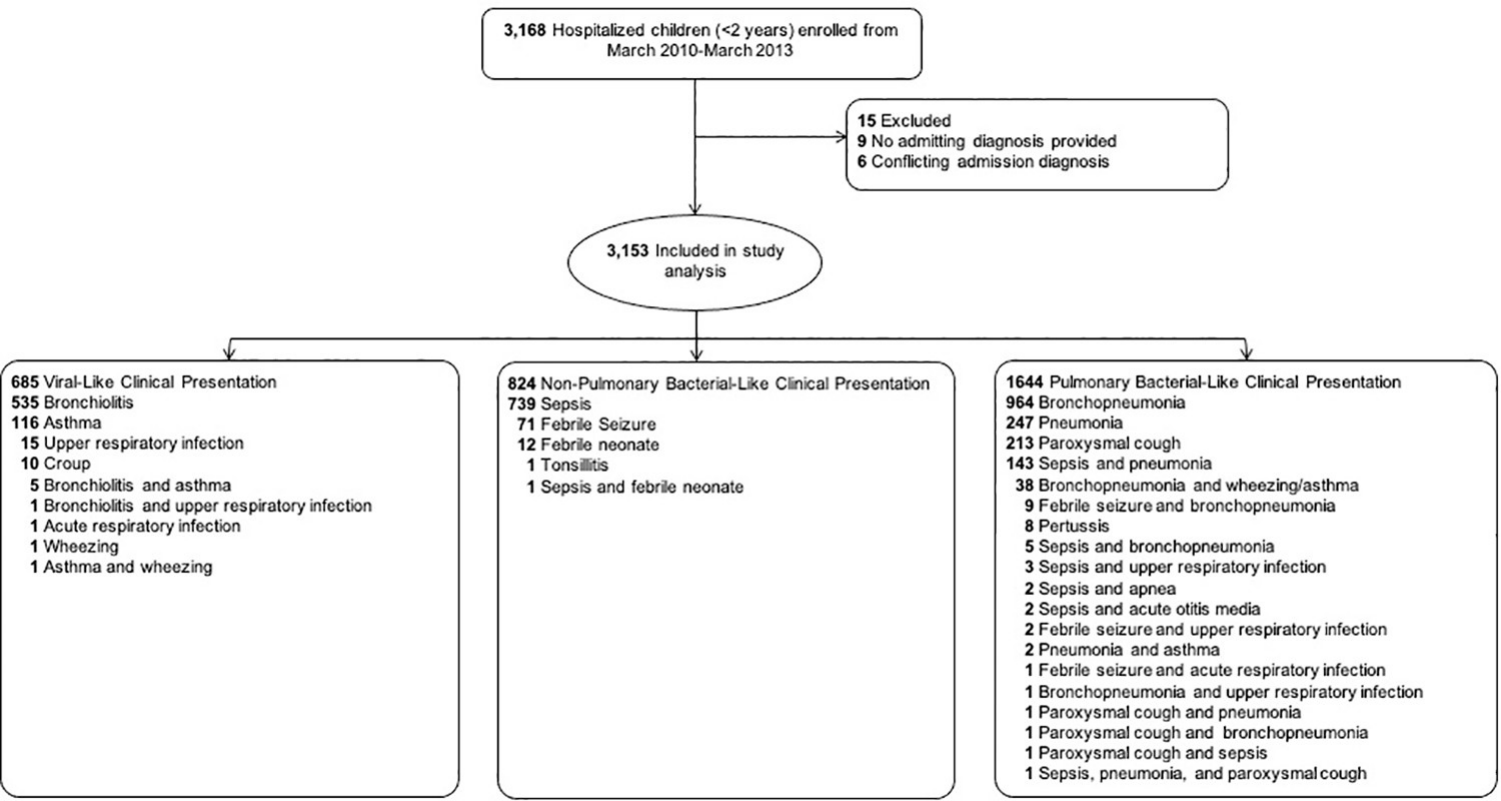
Consort diagram of hospitalized Jordanian children included in a cross-sectional cohort analysis assessing the association of admission diagnosis and antibiotic administration.

From March 2010 to March 2013, 3168 children were enrolled and 15 (0.5%) were excluded from this analysis: nine had no admission diagnosis and five had diagnoses that were not decipherable between suspected viral- and bacterial-like infection (**Fig 1**). The final study population represents 99% (*n* = 3153) of children enrolled into our parent study [11]. The most common primary admission diagnoses among children were bronchopneumonia (32%), rule-out sepsis (28%), and bronchiolitis (17%). Of the admission diagnoses, 2468 (78%) were classified as suspected bacterial-like infection (non-pulmonary = 824; pulmonary = 1644), indicating an empiric treatment, whereas 685 (22%) of diagnoses were classified as suspected viral-like infection (**Fig 1**).

### Comparison of demographic and illness characteristics by viral- and bacterial-like diagnoses

Of the 685 children with a suspected viral-like infection, 61% had RSV detected from their research laboratory specimen. These children were primarily male, had a median mean age of 5.8 months (SD: 4.7), and 89 (13.0%) had history of an underlying medical condition. Children with a viral-like infection most often reported cough (95%), wheezing (88%), and shortness of breath (85%); and were more likely have RSV detected by research RT-qPCR testing (**Table 1**) compared to children with suspected pulmonary or non-pulmonary bacterial-like infection. In contrast, children with suspected pulmonary or non-pulmonary bacterial-like infection had a higher frequency of fever (pulmonary: 57%; non-pulmonary: 71%), a longer hospitalization stay, more likely admitted to ICU. Children in the non-pulmonary bacterial-like infection group had the highest frequency of clinical laboratory confirmed bacterial infection (non-pulmonary: 11.2% vs. pulmonary: 2.9% and viral-like: 1.5%, *P*-value<0.001) with the majority being urinary tract infections (**Table 1**). Approximately 40% of our cohort had a parent or legal guardian report antibiotic use prior to hospitalization (44% viral-like, 51% pulmonary bacterial-like, 17% non-pulmonary bacterial-like).

**Table 1:** Demographic and illness characteristics of children under two years hospitalized between 2010–2013 in Amman, Jordan by Admission Presentation (*n* = 3,153).

Characteristic	Viral-Like Infection (*n* = 685)	Pulmonary Bacterial-Like Infection (*n* = 1644)	Non-Pulmonary Bacterial-Like Infection (*n* = 824)	All-Groups *P*-value^¥^^€^
Age, months—mean (SD)	5.8 (4.7)	7.4 (5.9)	2.6 (3.7)	**<0.001** ^¥^ ^€^
Sex, male—no.(%)	460 (67.2)	948 (57.7)	495 (60.1)	**<0.001** ^¥^ ^€^
Premature, <37 weeks	102 (14.9)	235 (14.3)	112 (13.6)	0.769
Birth weight, kg—mean (SD)	3 (0.7)	3 (0.7)	3 (0.6)	0.834
Underlying medical condition—no.(%)	89 (13.0)	238 (14.5)	45 (5.5)	**<0.001** ^€^
Maternal Education—no.(%)				
Primary education	271 (39.6)	676 (41.1)	328 (39.8)	0.878
Secondary education	306 (44.7)	713 (43.4)	374 (45.4)	
College	108 (15.8)	254 (15.5)	122 (14.8)	
Proximity of Residence—no.(%)				
Closest proximity	215 (31.4)	446 (27.2)	231 (28.1)	0.090
Moderate proximity	255 (37.2)	585 (35.7)	294 (35.7)	
Farthest proximity	215 (31.4)	610 (37.2)	298 (36.2)	
Antibiotic consumption prior to hospitalization—no. (%)	302 (44.1)	835 (50.8)	143 (17.4)	**<0.001** ^¥^ ^€^
Clinical Presentation—no. (%)				
Fever, >100.2 F	231 (33.7)	937 (57.0)	588 (71.4)	**<0.001** ^¥^ ^€^
Cough	648 (94.6)	1519 (92.4)	188 (22.8)	**<0.001** ^€^
Congestion	9 (1.3)	13 (0.8)	4 (0.5)	0.203
Runny nose	20 (2.9)	25 (1.5)	7 (0.9)	**0.006** ^¥^ ^€^
Wheezing	599 (87.5)	1055 (64.2)	91 (11.0)	**<0.001** ^¥^ ^€^
Shortness of breath	584 (85.3)	1140 (69.3)	100 (12.1)	**<0.001** ^¥^ ^€^
Hospitalization Factors—no. (%)				
ICU stay	40 (5.8)	151 (9.2)	88 (10.7)	**0.003** ^¥^ ^€^
Chest x-ray, abnormal	577/667 (86.5)	1425/1622 (87.9)	67/657 (10.2)	**<0.001** ^€^
Length of stay, days—mean (SD)	4 (3)	5 (4)	7 (4)	**<0.001** ^¥^ ^€^
Bacterial infection—no. (%)	10 (1.5)	47 (2.9)	92 (11.2)	**<0.001** ^¥^ ^€^
Blood culture, positive	3 (0.4)	11 (0.7)	23 (2.8)	**<0.001** ^€^
Urine culture, positive	7 (1.0)	36 (2.2)	75 (9.1)	**<0.001** ^€^
Cerebral spinal fluid, positive	0 (0.0)	1 (0.1)	3 (0.4)	0.078
Laboratory Results—no. (%)				
Respiratory Syncytial Virus	419 (61.2)	825 (50.2)	144 (17.5)	**<0.001** ^¥^ ^€^
Influenza	24 (3.5)	65 (4.0)	29 (3.5)	0.808
Adenovirus	93 (13.6)	270 (16.4)	111 (13.5)	0.074
Parainfluenza	36 (5.3)	94 (5.7)	45 (5.5)	0.899
Rhinovirus	283 (41.3)	622 (37.8)	327 (39.7)	0.268
Metapneumovirus	71 (10.4)	177 (10.8)	25 (3.0)	**<0.001** ^€^

Abbreviations: ICU, intensive care unit; Maternal education, [Primary education, grades 1–10; Secondary education, grades 11–12]; Proximity of Residence, [Closest proximity, zone 5 (i.e., Al-Qwesmeh); Moderate proximity, zones 1, 6 (i.e., Amman and Marka); Farthest proximity, zones 2–4, 7–36].

*P*-values were calculated using simple linear regression with robust standard errors for continuous and Pearson χ^2^ test for categorical variables, alpha set at <0.05.

^¥^ denotes p-value <0.05 for the pairwise comparison between viral-like infection and pulmonary bacterial-like infection.

^€^ denotes p-value <0.05 for the pairwise comparisons between viral-like infection and non-pulmonary bacterial-like infection.

### Antibiotic administration type

**Fig 2:**
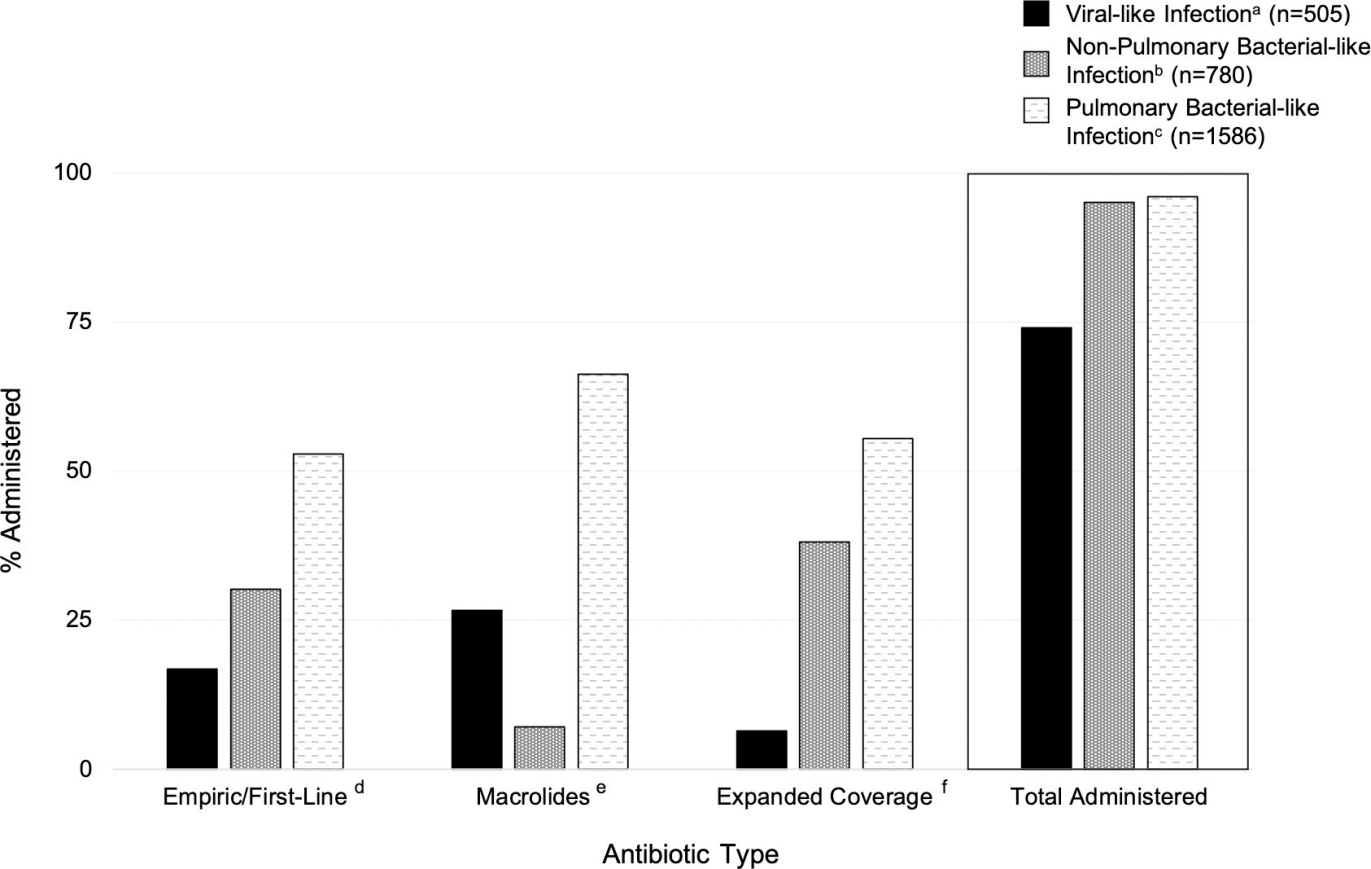
Percentage of antibiotics administered based on presenting clinical symptoms, by antibiotic type to children less than two hospitalized in Amman, Jordan. Footnote: ^a^ Viral-Like Infection—RSV, bronchiolitis, acute respiratory infection, upper respiratory infection, influenza, apnea, asthma, croup, and wheezing. ^b^ Non-Pulmonary Bacterial-Like Infection—pertussis, pneumonia, bronchopneumonia, and acute otitis media. ^c^ Pulmonary Bacterial-Like Infection—sepsis, febrile, febrile seizure, and tonsillitis. ^d^ Empiric/First-Line—ampicillin, gentamicin, cefotaxime, ceftriaxone, and cefuroxime. ^e^ Macrolides—erythromycin, clarithromycin, and azithromycin. ^f^ Expanded Coverage—vancomycin, ceftazidime, and amikacin.

Overall, 505 (74%) children with a suspected viral-like infection were administered at least one antibiotic during their hospitalization, of which 497 (98%) had no documentation of a concominant bacterial infection. Empiric/first-line antibiotics were the most common antibiotics administered during our study period (2166/2871 [75%]; with viral-like and pulmonary bacterial-like infections having similar administration percentages (73% and 72%, respectively). Whereas, non-pulmonary bacterial-like infections had the highest percentage with 84% given an antibiotic. Of the macrolides and expanded coverage antibiotics administered, macrolides were more often given to children with viral-like (27%) and pulmonary bacterial-like (66%) diagnoses, whereas expanded coverage antibiotics were often administered to those in the non-pulmonary bacterial-like group (38%) (**Fig 2**). Compared to children with a suspected non-pulmonary bacterial-like infection, children with a suspected viral-like and pulmonary bacterial-like infection were more frequently prescribed one antibiotic (75% viral-like and 63% pulmonary vs. 29% non-pulmonary) and had a shorter median therapy duration (viral-like and pulmonary bacterial-like: 4 days [IQR: 3–6 days]; non-pulmonary bacterial-like: 7 days [IQR: 4–8 days]).

Overall, both male and female children who presented to Al-Bashir Hospital with a suspected viral-like infection had an 88% and 86% lower prevalence of being administered an empiric/first-line antibiotic during their hospitalization (male, aPOR: 0.12; female, aPOR:0.14; *P*-value = <0.001) and an 95% and 91% lower prevalence of being administered an expanded coverage antibiotic (male, aPOR: 0.05; female, aPOR:0.09; *P*-value = <0.001) than those who presented with a suspected non-pulmonary bacterial-like infection (**Table 2**). Alternatively, males with a suspected viral-like infection had two-fold increased prevalence odds (aPOR: 2.40; 95% CI: 1.16, 4.96) of being administered a macrolide than males with a non-pulmonary bacterial-like infection. Whereas a significant association was not observed for macrolide administration in female children with a viral-like infection. Compared to non-pulmonary bacterial-like infections, both males and females with suspected pulmonary bacterial-like infection had a respective 19-fold and 16-fold increased prevalence odds of being administered a macrolide (male, aPOR: 19.0; female, aPOR:15.9; *P*-value = <0.001, **Table 2**).

**Table 2:** Association of clinical suspicion for a viral infection and the administration of antibiotics to children hospitalized between 2010–2013 in Amman, Jordan, Stratified by sex.

	Empiric/First-Line ^a^			Macrolides ^b^			Expanded Coverage ^c^		
Admission Presentation	aPOR^d^	95% CI	*P*-value	aPOR^d^	95% CI	*P*-value	aPOR^d^	95% CI	*P*-value
Males (*n* = 1899)									
Bacterial-Like									
Non-Pulmonary	1.00	Ref	Ref	1.00	Ref	Ref	1.00	Ref	Ref
Pulmonary	1.13	0.63, 2.04	0.686	19.0	8.8, 41.0	<0.001	1.98	0.96, 4.05	0.063
Viral-Like	0.12	0.07, 0.20	<0.001	2.40	1.16, 4.96	0.018	0.05	0.02, 0.14	<0.001
Females (*n* = 1249)									
Bacterial-Like									
Non-Pulmonary	1.00	Ref	Ref	1.00	Ref	Ref	1.00	Ref	Ref
Pulmonary	1.22	0.60, 2.51	0.580	15.9	6.57, 38.41	<0.001	1.48	0.64, 3.42	0.360
Viral-Like	0.14	0.07, 0.26	<0.001	1.50	0.63, 3.57	0.357	0.09	0.03, 0.25	<0.001

Footnote: P-values were based on a two-tailed probability and a significance level set at alpha <0.05.

^a^ Empiric/First-line—ampicillin, gentamicin, cefotaxime, ceftriaxone, and cefuroxime.

^b^ Macrolides—erythromycin, clarithromycin, and azithromycin.

^c^ Expanded Coverage—vancomycin, ceftazidime, and amikacin.

^d^Models were adjusted for age, underlying medical condition, region of residence, and maternal education.

### Research molecular testing and antibiotic use

When comparing pulmonary bacterial-like admission diagnosis to research RT-qPCR viral results, there was a 34% (sensitivity) probability the admitting clinician would diagnose a child with RSV as a suspected viral-like infection given the child was RSV-positive based on their RT-qPCR results (**Table 3**). Comparatively, among children who were RSV-negative, 76% were diagnosed with a suspected pulmonary bacterial-like infection upon admission (specificity). However, the sensitivity and specificity between non-pulmonary bacterial-like and suspected viral-like admission diagnoses and RT-qPCR RSV results had better diagnostic accuracy (sensitivity: 74%; specificity: 72%). When further evaluating the sensitivity and specificity comparing virus-positive to virus-negative, our results were similar (**Table 3**).

**Table 3:** Performance comparison of admission diagnosis to RT-qPCR-confirmed respiratory syncytial virus among children hospitalized in Amman, Jordan, 2010–2013.

	RT-qPCR Respiratory Syncytial Virus				RT-qPCR Any Virus Positive			
	Viral-Like vs. Non-Pulmonary		Viral-Like vs. Pulmonary		Viral-Like vs. Non-Pulmonary		Viral-Like vs. Pulmonary	
Performance Characteristics	%	95% CI	%	95% CI	%	95% CI	%	95% CI
Sensitivity^a^	74.4	70.6, 78.0	33.7	31.1, 36.4	55.1	52.1, 58.0	30.3	28.3, 32.3
Specificity^b^	71.9	68.9, 74.7	75.5	72.8, 78.0	83.8	79.7, 87.4	77.4	71.9, 82.3
Positive Predictive Value	61.2	57.4, 64.8	61.2	57.4, 64.8	91.1	88.7, 93.1	91.1	88.7, 93.1
Negative Predictive Value	82.5	79.8, 85.1	49.8	47.4, 52.3	38.2	34.9, 41.6	12.7	11.1, 14.4

^a^Sensitivity denotes the probability of having a suspected viral-like infection (i.e., RSV, bronchiolitis, acute respiratory infection, upper respiratory infection, influenza, apnea, asthma, croup, and wheezing) given, the patient is positive for a virus.

^b^Specificity denotes the probability of having a suspected bacterial-like infection (i.e., non-pulmonary: Sepsis, febrile, febrile seizure, tonsillitis; pulmonary: Pertussis, pneumonia, tonsillitis, bronchopneumonia, and acute otitis media) given, the patient is negative for a virus.

## Discussion

In our study of Jordanian children hospitalized with fever and/or acute respiratory symptoms over 90% of the cohort had an antibiotic administered during their hospitalization course and nearly half of the children received more than one antibiotic. When we evaluated antibiotic administration based on admission diagnosis, children with suspected viral-like infection diagnoses had a reduced prevalence of being administered an empiric/first-line or expanded coverage antibiotic; and specifically males were more likely to be administered a macrolide. Although we observed a reduced prevalence of antibiotics in children with suspected viral-like infection, we found discriminating between a viral-like and pulmonary bacterial-like infections with clinical presentation alone is insufficient in correctly determining causative pathogens that warrant empiric antibiotic use; thus, placing an emphasis on the importance of point-of-care diagnostic testing.

Previous studies in Jordan examining antibiotic administration in an inpatient setting have been limited. One study among 5,829 children with acute respiratory infections at an ambulatory care setting, found 69% of children were prescribed unwarranted antibiotics [9]. Of note, this finding was after international guidance had been initiated to improve antibiotic prescribing [9,13]. Similarly, in a retrospective surveillance study of children under 14 years who sought medical attention from military outpatient emergency clinics, reported 85% of encounters resulted in a prescribed antibiotic [8]. We also found nearly a quarter of our cohort were still administered an antibiotic with only viral-like admission diagnoses. Other studies from the Middle East, including Egypt, Kuwait, and Saudi Arabia have reported consistent prescribing thresholds for upper respiratory infections [13]. These studies provide valuable context in understanding inappropriate use of antibiotics in Jordan and other Middle Eastern countries; however, reports of prescription proportions is nonspecific when rapid diagnostic testing in these settings for respiratory viruses are not readily available. To overcome this barrier, we assessed antibiotic practices from the perspective of clinical presentation, which may warrant empiric therapy. When we categorized children by their admitting diagnosis, we found that children with a suspected viral-like infection had an decreased prevalence of being administered an empiric/first-line and expanded coverage antibiotic compared to children with suspected non-pulmonary bacterial-like infection. But found males with a suspected viral-like infection were more likely to be given a macrolide. Nonethesless, when we compared the admission diagnoses to RT-qPCR testing, we found nearly two-thirds of children with RSV may have been misclassified into a suspected pulmonary bacterial-like diagnosis. In the United States, reports of serious bacterial co-infections with RSV have been low [14,15], therefore in this cohort there may be implications for waiting to administer empiric antibiotics to children with clinical diagnoses consistent with RSV. Our findings underscore the challenges that are encountered when managing patients with respiratory viruses that mimic the symptomology of bacterial pathogens without diagnostic resources. Additional studies are imperative to determine solutions to overcome the prescribing barriers in Jordan.

Although we cannot assess the specific prescribing barriers at the provider-level, possible mechanisms attributed to the therapeutic decisions of pediatricians in Jordan were hypothesized. Besides the lack of point-of-care diagnostics and other laboratory resources, parental pressures may influence the clinician’s likelihood to administer antibiotics to a hospitalized child. This perspective is multifaceted, but include the parent’s education levels, beliefs, and socioeconomic status; and the provider’s antibiotic administration preferences [16,17]. For example, a recent study evaluating parental attitude towards antibiotic use in Jordan found 84% of parents believe a medication is necessary in treating illnesses [17]. Another major barrier in antimicrobial stewardship is in Jordan antibiotics are available over-the-counter from a pharmacy, even without a prescription [18]. Although there are regulations in Jordan prohibiting the dispensing of antibiotics without a prescription, several studies have reported this is not strictly enforced [18–20]. Finally, there may be a continued belief from Jordanian clinicians that there is a prophylactic antibiotic use is warranted for the prevention of a secondary bacterial infections [8]. In our study we attempted to overcome this barrier by categorizing our exposure variable (admission diagnosis). Thus, understanding both provider and parental perceptions through surveys and educational campaigns, along with laboratory diagnostics are important to address antibiotic prescribing patterns in Jordan.

Studies in Australia, Brazil, and the Netherlands have evaluated the impact of laboratory testing for RSV and antibiotic prescriptions [3,21,22]. All results from these studies showed an improvement in antibiotic prescribing patterns. Specifically in a study conducted by O’Callaghan et al. reported that there was reduced antibiotic use in pediatric patients who were positive for a respiratory virus [3]. Similarly, in an influenza rapid diagnostic study conducted in a rural community in Thailand, found a 59% decreased odds of being prescribed an antibiotic when influenza was detected from a rapid test [23]. Despite the improvements these countries have had in antibiotic prescribing, routine laboratory testing in the United States for bronchiolitis (RSV) is not recommended [24]. Although routine laboratory testing is not recommended for RSV in the United States, the healthcare settings have antimicrobial stewardship programs that are tracking prescribing/administration patterns and have formulary restrictions based on antimicrobial resistance. In countries with limited laboratory testing and antimicrobial stewardship programs, point-of-care testing paired with clinical suspicion may aid providers in determining whether empiric antibiotic treatment is warranted and help limit misclassification of clinical diagnoses.

Additional studies are vital to understand whether point-of-care viral diagnostic testing among this Jordanian pediatric population would decrease antibiotic use when they are not clinically indicated.

The strengths of our study include a large pediatric cohort and the inclusion of research molecular laboratory-confirmed RSV detection. During our study period, we were able to capture approximately 50–60% of hospitalizations among children in Amman were admitted at Al-Bashir Hospital [10]. In addition, we systematically performed molecular viral testing on all children enrolled into our study, further limiting indication bias. Although we had several strengths, we also had some notable limitations worth mentioning. First, ascertainment of concomitant bacterial infections was collected via medical chart reviews when a positive laboratory culture result was identified. This limitation may have introduced non-differential misclassification of bacterial infections and may have overestimated the true association of the outcome. However, we do not believe this largely impacted our study as many studies have shown bacterial infections co-infected with RSV to be less than 10 percent [14,15]. Second, we only evaluated antibiotic administration patterns among hospitalized children and did not include other healthcare settings, such as emergency departments or ambulatory clinics. Therefore, the antibiotic patterns identified in our study may not be representative of the practices across different healthcare settings. Third, nearly 40% of children reported receiving an antibiotic before admission; thus, this may have underestimated the isolation of bacteria from clinical cultures. Finally, we calculated the sensitivity, specificity, negative, and positive predictive values of admission diagnoses to research molecular testing results. Negative and positive predictive values should be interpreted with caution as they are influenced by the prevalence of RSV circulating in the community; and sensitivity and specificity measures do not reflect bacterial respiratory infections. Furthermore, our study was designed as a viral surveillance to determine the burden of respiratory viruses causing acute respiratory illness.

Our cross-sectional study evaluating the relationship between clinical presentation and antibiotic administration among children hospitalized in Amman, Jordan indicated that children with a suspected viral-like admission diagnosis have a lower prevalence of being administered empiric/first-line and expanded coverage antibiotics as those hospitalized with a suspected non-pulmonary bacterial-like infection. However, out of those patients that presented with a suspected pulmonary bacterial-like infection and were administered an empiric antibiotic, only 37% were RSV-negative. These results highlight the need for developing antibiotic prescribing interventions for Jordan and the rest of the Middle Eastern countries. To fully understand the complexity of the problem, future studies should evaluate the types of antibiotics prescribed and the course duration. Assessments of provider barriers on antibiotic prescribing practices would also be useful in developing sustainable guidelines and interventions for antibiotic stewardship.

## Supporting information

S1 Dataset (CSV)
